# Venous disease classifications and generic and disease-specific quality of life questionnaires: which, why, and when to use?

**DOI:** 10.1590/1677-5449.190114

**Published:** 2019-11-21

**Authors:** Vanessa Prado dos Santos, André Brito Queiroz

**Affiliations:** 1 Universidade Federal da Bahia – UFBA, Instituto de Humanidades Artes e Ciências Professor Milton Santos, Salvador, BA, Brasil.; 2 Complexo Hospitalar Universitário Professor Edgard Santos – UFBA, Salvador, BA, Brasil.

The multiplicity of acronyms and concepts related to venous disease is an indication of the complexity involved. The term chronic venous disease (CVD) encompasses the many different signs and symptoms of venous disease.^1^^,^ 2 In turn, chronic venous insufficiency (CVI) refers to disease of greater severity, with CEAP classes from C3 to C6, taking in presentations ranging from edema to ulceration.^1^^,^ 2 However, some authors reserve the term CVI for cases with damage to the skin and subcutaneous tissues, defining CVI as CEAP classes C4 to C6.^3^ A number of different venous disease classifications have been proposed to help with diagnosis, treatment, and follow-up. The CEAP classification, revised in 2004, covers the many different signs of venous involvement, within the dimensions C (clinical signs); E (etiologic classification); A (anatomic distribution), and P (pathophysiologic dysfunction)^4^^,^ 5 (Table 1). The authors of this classification discuss the term “disease”, proposing that the lower CEAP classes should be referred to as chronic venous disorders.^5^ The Venous Clinical Severity Score (VCSS), revised in 2010, is used to monitor the symptoms of CVD and measure its severity and does not include telangiectasies or reticular veins^6^^,^ 7 (Table 2). Clinical CVD classifications are used to guide diagnostic investigation, monitor disease progression, and evaluate treatment results. Notwithstanding, a patient who has been classified as CEAP 5 may exhibit clinical improvement, but remain at the same classification after treatment despite this improvement. Considering the complexity of the venous disease, it is clear that there are multiple different situations in which we need additional criteria to evaluate disease progression. The literature proposes employing the VCSS and CEAP in conjunction and suggests that quality of life (QoL) questionnaires should also be used.^7^ Regardless of whether the case is a disorder, disease, or insufficiency, the symptoms and signs of a compromised venous system impact on people’s QoL.^8^^,^ 9 Concern with aspects related to QoL is a growing part of the healthcare debate. The World Health Organization (WHO) defines QoL as “an individual's perception of their position in life in the context of the culture and value systems in which they live and in relation to their goals, expectations, standards and concerns.”^10^ So, in addition to the classifications already mentioned, what QoL questionnaires have been proposed for venous disease? In healthcare, QoL may be related to general condition and studied using generic questionnaires or it may be related to certain diseases and assessed using disease-specific instruments.^8^^,^ 9 ^,^ 11 The World Health Organization Instrument to Assess Quality of Life (WHOQOL) and 36-item Short-Form Health Survey (SF-36) questionnaires are both generic instruments for studying QoL, while the Chronic Venous Insufficiency Questionnaire (CIVIQ), the Venous Insufficiency Epidemiological and Economic Study/Quality of Life-Symptoms (VEINES/QoL-Sym), the Aberdeen Varicose Vein Questionnaire (AVVQ), and the Charing Cross Venous Ulceration Questionnaire (CXVUQ) are all specific to QoL in CVD^8^^,^ 9 ^,^ 12 ^,^ 13 (Table 3). The WHOQOL-100 has 100 questions distributed across six domains: physical health, psychological, level of independence, social relations, environment, and spirituality/religion/personal beliefs.^10^^,^ 12 ^,^ 14 The SF-36 is a generic questionnaire that assesses physical functioning, role-physical, bodily pain, general health, vitality, social functioning, role-emotional, and mental health and also compares current health with health 1 year previously.^15^^,^ 16 The generic questionnaires revealed a need to assess the impact on QoL of specific diseases. The CIVIQ, VEINES, AVVQ, and CXVUQ are questionnaires specifically designed for venous disease.^8^^,^ 9 The CIVIQ and the VEINES/QoL-Sym assess venous disease in general and the AVVQ and the CXVUQ deal with specific aspects of CVD.^8^^,^ 9 The CIVIQ-20 focuses on the physical, psychological, social, and pain dimensions, with questions that cover daily activities, sleeping, pain, and irritability.^8^^,^ 9 ^,^ 17 The VEINES/QoL-Sym questionnaire comprises two scales: the VEINES-QoL, which covers QOL in CVD, and the VEINES-Sym, which evaluates CVD symptoms^18^, and has been translated and adapted for use in Brazil.^11^ The AVVQ was developed to assess QOL in patients with varicose veins and includes a diagram of the lower limbs on which the patient draws.^8^^,^ 9 ^,^ 19 ^,^ 20 The CXVUQ is designed to assess QOL in patients with active venous ulcers.^21^^,^ 22 Specific questionnaires provide more detailed information when used in conjunction with generic ones.^8^ While it may be difficult to apply a clinical classification and administer both a generic and a specific questionnaire in routine practice, knowing all of the different classification and questionnaires puts us in a position to decide which to adopt to the benefit of our practice. Currently, clinical classifications are the most frequently employed when discussing CVD and have demonstrated associations with QoL.^8^ Specific QoL questionnaires have been translated, adapted, and validated for use in Brazil. If we chose to employ these instruments, the patient can fill out a QoL questionnaire in the waiting room and we can apply one of the clinical classifications during the consultation. We will thus be able to monitor the venous diseases that we meet in our routine practice and assess their impact on our patients’ daily lives.

**Table 1:** Revised Clinical-Etiology-Anatomy-Pathophysiology (CEAP) Classification.^5^

**C (Clinical)**	**E (Etiology)**	**A (Anatomy)**	**P (Pathophysiology)**
C0: no visible or palpable signs of venous disease	Ec: congenital	As: superficial veins	Pr: reflux
C1: telangiectasias (< 1 mm) or reticular veins (≥ 1 mm e < 3 mm)	Ep: primary	Ap: perforating veins	Po: obstruction
C2: varicose veins (diameter ≥ 3 mm) C3: edema	Es: secondary (post-thrombotic)	Ad: deep veins	Pr,o: reflux and obstruction
C4: secondary alterations to skin and subcutaneous tissues C4a: eczema or pigmentation C4b: lipodermatosclerosis or white atrophy	En: without identified venous etiology	An: venous location not determined	Pn: unidentified venous pathophysiology
C5: healed venous ulcer C6: active venous ulcer			

CVD: Chronic Venous Disease.

**Table 2:** Revised Venous Clinical Severity Score (VCSS).^7^

**Clinical characteristic**	**None (0)**	**Mild (1)**	**Moderate (2)**	**Severe (3)**
Pain (or other discomfort: presumes venous origin)	-	Occasional	Daily (does not prevent activities)	Daily (limits activities)
Varicose veins (≥ 3 mm when standing)	-	Few (scattered)	Confined to calf or thigh	Involves calf and thigh
Venous edema (presumes venous origin)	-	Limited to foot and ankle	Extends above ankle, but below knee	Extends to knee and above
Skin pigmentation (presumes venous origin)	None or focal	Limited to perimalleolar area	Diffuse over lower third of calf	Above lower third of calf
Inflammation (erythema, eczema, dermatitis)	-	Limited to perimalleolar area	Diffuse over lower third of calf	Above lower third of calf
Induration (fibrosis, hypodermitis, white atrophy, lipodermatosclerosis)	-	Limited to perimalleolar area	Diffuse over lower third of calf	Above lower third of calf
Active ulcer number	0	1	2	≥ 3
Active ulcer duration	N/A	< 3 months	> 3 months, but < 1 year	Unhealed > 1 year
Active ulcer size	N/A	Diameter < 2 cm	Diameter 2-6 cm	Diameter > 6 cm
Use of compression therapy	(0) not used	(1) intermittent use	(2) compression therapy most days	(3) full compliance: compression therapy

**Table 3:** Summary of generic and CVD-specific quality of life questionnaires.

**Questionnaire**	**Type of instrument/target population**	**Number of questions/abbreviated versions**	**References for Portuguese translations of instruments**
WHOQOL	Generic	WHOQOL-100 items and WHOQOL-Bref with 26 items	Fleck et al.^14^
SF-36	Generic	SF-36 items; SF-12 (abbreviated to 12 items)	Ciconelli et al.^15^
CIVIQ	Specific/patients with CVD	CIVIQ-20 items and CIVIQ-14 (abbreviated to 14 items)	Leal & Mansilha^8^
VEINES	Specific/ patients with CVD	Two scores: VEINES/Sym (10 items) and VEINES/QoL (25 items)	Moura et al.^11^
AVVQ	Specific/patients with lower limb varicose veins	13 items/12 questions and a diagram for drawing varicose veins	Leal et al.^20^
CXVUQ	Specific/patients with venous ulcer	21 items/four domains	Couto et al.^22^

AVVQ: Aberdeen Varicose Vein Questionnaire; CIVIQ: Chronic Venous Insufficiency Questionnaire; CXVUQ: Charing Cross Venous Ulceration Questionnaire; CVD: chronic venous disease; SF-36: 36-item Short-Form Health Survey; VEINES: Venous Insufficiency Epidemiological and Economic Study; WHOQOL: World Health Organization Instrument to Assess Quality of Life.
