# Supplementary material for: Venous disease classifications and generic and disease-specific quality of life questionnaires: which, why, and when to use?
Source: J Vasc Bras. 2019 Nov 21;18:e20190114. doi: 10.1590/1677-5449.190114 (PMC6880618; doi:10.1590/1677-5449.190114)
Supplement: Translation

# Translation: Classificações e questionários de qualidade de vida na doença venosa: quais são, por que e quando utilizar?

**Authors:** Vanessa Prado dos Santos, André Brito Queiroz

**Affiliations:** 1 Universidade Federal da Bahia – UFBA, Instituto de Humanidades Artes e Ciências Professor Milton Santos, Salvador, BA, Brasil.; 2 Complexo Hospitalar Universitário Professor Edgard Santos – UFBA, Salvador, BA, Brasil.

A complexidade que envolve a doença venosa pode ser observada na multiplicidade de siglas e conceitos utilizados. O termo doença venosa crônica (DVC) abrange os diversos sinais e sintomas da doença venosa^1^^,^ 2. Já insuficiência venosa crônica (IVC) remonta a doença de maior gravidade, quando a classificação CEAP está entre C3-C6, desde o edema até a ulceração^1^^,^ 2. No entanto, alguns autores utilizam o termo IVC apenas quando há alterações de pele e tecido subcutâneo, considerando IVC as classes entre C4 e C6^3^. Para estudar melhor a doença venosa, seu diagnóstico, tratamento e acompanhamento, foram propostas algumas classificações. A classificação CEAP, revisada em 2004, inclui os diversos sinais de comprometimento venoso e traz as dimensões C (*clinical signs*); E (*etiologic classification*); A (*anatomic distribution*) e P (*pathophysiologic dysfunction*)^4^^,^ 5 (Tabela 1). Os autores discutem o termo “doença”, propondo que as classificações iniciais do CEAP sejam denominadas desordens venosas crônicas (*chronic venous disorders*)^5^. O *Venous Clinical Severity Score* (VCSS), revisado em 2010, é utilizado para acompanhar os sintomas e mensurar a gravidade da DVC e não inclui no escore telangiectasias e veias reticulares^6^^,^ 7 (Tabela 2). As classificações clínicas da DVC são utilizadas para nortear a investigação diagnóstica, acompanhar a evolução da doença e avaliar os resultados do tratamento. No entanto, um doente classificado como CEAP 5 pode, apesar da sua melhora clínica, manter-se na mesma classificação inicial após o tratamento. Pensando na complexidade da doença venosa, percebemos a multiplicidade de situações em que precisamos de outros critérios para avaliar a evolução da doença. A literatura propõe a utilização conjunta do VCSS e do CEAP, e sugere que sejam associados questionários de qualidade de vida (QV)^7^. Seja desordem, doença ou insuficiência, o termo aplicado, os sintomas e sinais decorrentes do comprometimento do sistema venoso influenciam na QV das pessoas^8^^,^ 9. A preocupação com aspectos relacionados à QV vem crescendo nas discussões do campo da saúde. A Organização Mundial da Saúde (OMS) definiu QV como “a percepção do indivíduo de sua posição na vida no contexto da cultura e sistema de valores nos quais ele vive e em relação aos seus objetivos, expectativas, padrões e preocupações”^10^. Sendo assim, além das classificações mencionadas, quais seriam os questionários de QV propostos para a doença venosa? A QV em saúde pode estar relacionada a condições gerais, estudadas por meio de questionários genéricos, ou a algumas doenças, avaliada por meio de instrumentos específicos^8^^,^ 9 ^,^ 11. Os questionários *World Health Organization Instrument to Assess Quality of Life* (WHOQOL) e *36-item Short-Form Health Survey* (SF-36) são instrumentos genéricos para estudar a QV, enquanto o *Chronic Venous Insufficiency Questionnaire* (CIVIQ), o *Venous Insufficiency Epidemiological and Economic Study/Quality of Life-Symptoms* (VEINES/QoL-Sym), o *Aberdeen Varicose Vein Questionnaire* (AVVQ) e o *Charing Cross Venous Ulceration Questionnaire* (CXVUQ) são específicos para a QV na DVC^8^^,^ 9 ^,^ 12 ^,^ 13 (Tabela 3). O WHOQOL-100 contém 100 questões distribuídas em seis domínios: físico, psicológico, nível de independência, relações sociais, meio ambiente e espiritualidade/religiosidade/crenças pessoais^10^^,^ 12 ^,^ 14. O SF-36 é um questionário genérico que avalia capacidade funcional, aspectos físicos, dor, estado geral de saúde, vitalidade, aspectos sociais, aspectos emocionais e saúde mental, além de comparar a saúde atual e há 1 ano^15^^,^ 16. A partir dos questionários genéricos, foi identificada a necessidade de avaliar o impacto de doenças específicas na QV. Os questionários específicos para a doença venosa são: CIVIQ, VEINES, AVVQ e CXVUQ^8^^,^ 9. O CIVIQ e o VEINES/QoL-Sym avaliam a doença venosa de maneira geral, e o AVVQ e o CXVUQ tratam de aspectos específicos da DVC^8^^,^ 9. O CIVIQ-20 enfoca as dimensões física, psicológica, social e dor, com perguntas que incluem atividades diárias, sono, dor e irritabilidade^8^^,^ 9 ^,^ 17. O questionário VEINES/QoL-Sym é composto de dois escores: o VEINES-QoL, que trata da QV na DVC, e o VEINES-Sym, que diz respeito aos sintomas de DVC^18^, tendo sido traduzido e adaptado no Brasil^11^. O AVVQ foi desenvolvido para avaliar a QV em doentes com varizes e conta com um diagrama dos membros inferiores, para que o paciente as desenhe^8^^,^ 9 ^,^ 19 ^,^ 20. O CXVUQ é voltado para avaliar a QV em doentes com úlcera venosa ativa^21^^,^ 22. Questionários específicos fornecem informações mais detalhadas quando utilizados em conjunto com os genéricos^8^. Embora aplicar uma classificação clínica, acrescentando um questionário de QV genérico e um específico, possa dificultar o uso rotineiro desses instrumentos, conhecer as diferentes classificações e questionários nos permite avaliar qual adotar em benefício da nossa prática. Atualmente, as classificações clínicas são as mais utilizadas quando se discute DVC, mostrando associação com a QV^8^. No Brasil, questionários específicos de QV foram traduzidos, adaptados e validados. Se pensarmos em utilizar esses instrumentos, o questionário de QV pode ser respondido pelo doente enquanto aguarda o atendimento, e durante a consulta já aplicamos uma das classificações clínicas. Ou seja, temos a possibilidade de acompanhar a doença venosa, que tratamos na nossa rotina, conhecendo o impacto que ela tem no dia a dia dos nossos pacientes.

**Tabela 1:** Classificação*Clinical-Etiology-Anatomy-Pathophysiology* (CEAP) revisada^5^.

**C (Clinica)**	**E (Etiologia)**	**A (Anatomia)**	**P (Fisiopatologia)**
C0: sem sinais visíveis ou palpáveis de doença venosa	Ec: congênita	As: veias superficiais	Pr: refluxo
C1: telangiectasias (< 1 mm) ou veias reticulares (≥ 1 mm e < 3 mm)	Ep: primária	Ap: veias perfurantes	Po: obstrução
C2: veias varicosas (diâmetro ≥ 3 mm) C3: edema	Es: secundária (pós-trombótica)	Ad: veias profundas	Pr,o: refluxo e obstrução
C4: alterações de pele e tecido subcutâneo secundárias a DVC C4a: eczema ou pigmentação C4b: lipodermatoesclerose ou atrofia branca	En: sem causa venosa identificada	An: localização venosa não identificada	Pn: fisiopatologia venosa não identificada
C5: úlcera venosa cicatrizada C6: úlcera venosa ativa			

DVC: Doença Venosa Crônica.

**Tabela 2:** Escore*Venous Clinical Severity Score* (VCSS) revisado^7^.

**Característica clínica**	**Não (0)**	**Leve (1)**	**Moderado (2)**	**Grave (3)**
Dor (ou outro desconforto de origem venosa presumida)	-	Ocasional	Diária (não limita atividades)	Diária (limitando as atividades)
Veias varicosas (≥ 3 mm em ortostase)	-	Poucas (isoladas)	Restrita à região da panturrilha ou da coxa	Na região da panturrilha e da coxa
Edema venoso (origem venosa presumida)	-	Limitado às áreas do pé e tornozelo	Extensão acima do tornozelo, mas abaixo do joelho	Atinge o joelho e acima dele
Pigmentação da pele (origem venosa presumida)	Nenhuma ou focal	Limitada à região perimaleolar	Difusa sobre o terço inferior da panturrilha	Acima do terço inferior da panturrilha
Inflamação (eritema, eczema dermatite)	-	Limitada à região perimaleolar	Difusa sobre o terço inferior da panturrilha	Acima do terço inferior da panturrilha
Enduração (fibrose, hipodermite, atrofia branca, lipodermatoesclerose)	-	Limitada à região perimaleolar	Difusa sobre o terço inferior da panturrilha	Acima do terço inferior da panturrilha
Número de úlceras ativas	0	1	2	≥ 3
Duração da úlcera ativa	N/A	< 3 meses	> 3 meses, mas < 1 ano	Não cicatriza > 1 ano
Tamanho da úlcera ativa	N/A	Diâmetro < 2 cm	Diâmetro 2-6 cm	Diâmetro > 6 cm
Uso de terapia compressiva	(0) não usa	(1) uso intermitente	(2) terapia compressiva na maioria dos dias	(3) adesão completa a terapia compressiva

**Tabela 3:** Resumo dos questionários de qualidade de vida genéricos e específicos para DVC.

**Questionário**	**Tipo de instrumento/população alvo**	**Número de questões/versões abreviadas**	**Referência dos instrumentos traduzidos para o português**
WHOQOL	Genérico	WHOQOL-100 itens e WHOQOL-Bref com 26 itens	Fleck et al.^14^
SF-36	Genérico	SF-36 itens; SF-12 (abreviado: 12 itens)	Ciconelli et al.^15^
CIVIQ	Específico/pacientes com DVC	CIVIQ-20 itens e CIVIQ-14 (abreviado: 14 itens)	Leal & Mansilha^8^
VEINES	Específico/ pacientes com DVC	Dois escores: VEINES/Sym (10 itens) e VEINES/QoL (25 itens)	Moura et al.^11^
AVVQ	Específico/pacientes com varizes de membros inferiores	13 itens/12 perguntas e um diagrama para desenhar as veias varicosas	Leal et al.^20^
CXVUQ	Específico/pacientes com úlcera venosa	21 itens/quatro domínios	Couto et al.^22^

AVVQ: *Aberdeen Varicose Vein Questionnaire*; CIVIQ: *Chronic Venous Insufficiency Questionnaire*; CXVUQ: C*haring Cross Venous Ulceration Questionnaire*; DVC: DoençaVenosaCrônica; SF-36: *36-item Short-Form Health Survey*; VEINES: *Venous Insufficiency Epidemiological and Economic Study*; WHOQOL: *World Health Organization Instrument to Assess Quality of Life*.
